# Case Report: Tumor-to-tumor metastasis with prostate cancer metastatic to lung cancer: the first reported case

**DOI:** 10.3389/fonc.2023.1238331

**Published:** 2023-08-18

**Authors:** Shiyue Liu, Hong Li, Youhong Dong, Dongdong Zhang

**Affiliations:** ^1^ Department of Oncology, Xiangyang No.1 People`s Hospital, Hubei Univeristy of Medicine, XiangYang, Hubei, China; ^2^ Department of Rehabilitation Medicine, Xiangyang No.1 People`s Hospital, Hubei Univeristy of Medicine, XiangYang, Hubei, China

**Keywords:** tumor-to-tumor metastasis, prostate cancer, lung cancer, adenocarcinoma, metastases

## Abstract

Tumor-to-tumor metastasis (TTM) occurs rarely in tumor progression, but this event has significant clinical implications. Although the impact of TTM on patient prognosis and survival has been increasingly recognized, understanding of TTM biology and treatment is limited. Prostate cancer is among the most common malignancies threatening male health. Prostate cancer can potentially metastasize to primary lung Cancer; however, this is an exceedingly rare event. We here report for the first time a case of TTM from a prostate cancer to a coexisting primary lung cancer.

## Introduction

Tumor-to-tumor metastasis (TTM), also known as tumor collision, is a rare event in which one primary tumor metastasizes to another distinct primary tumor (1). The TTM incidence is 0.2%–3.5% in autopsy series, which indicates its rarity (2).

The TTM pathophysiology is not yet completely understood, but several theories have been proposed. One theory suggests that circulating tumor cells from the primary tumor lodge in the secondary tumor’s vasculature and grow within the new microenvironment (3, 4). According to another theory, the secondary tumor creates a favorable environment allowing the growth of these circulating tumor cells (5, 6).

TTM diagnosis can be challenging because of its rarity and the similarity between the metastatic lesion and the primary tumor. Careful histopathological examination and immunohistochemical staining are necessary for differentiating a metastatic lesion and a second primary tumor. TTM can have substantial clinical implications, thereby affecting the disease stage and treatment approach. Awareness about TTM is crucial for accurate diagnosis and appropriate management.

We here present a case of a patient with a suspicious lung mass. Pathological examination revealed that the lung mass had two components: a lung adenocarcinoma and a prostate cell carcinoma. Subsequent imaging and pathological results confirmed the presence of multiple primary tumors in the patient, with the evidence of prostate cancer metastasizing to lung cancer. A personalized treatment plan was developed for the patient, which has thus far exhibited promising results. This case highlights the diagnostic challenges and clinical implications of TTM and offers some guidance for similar cases.

## Case presentation

**Figure 1:**
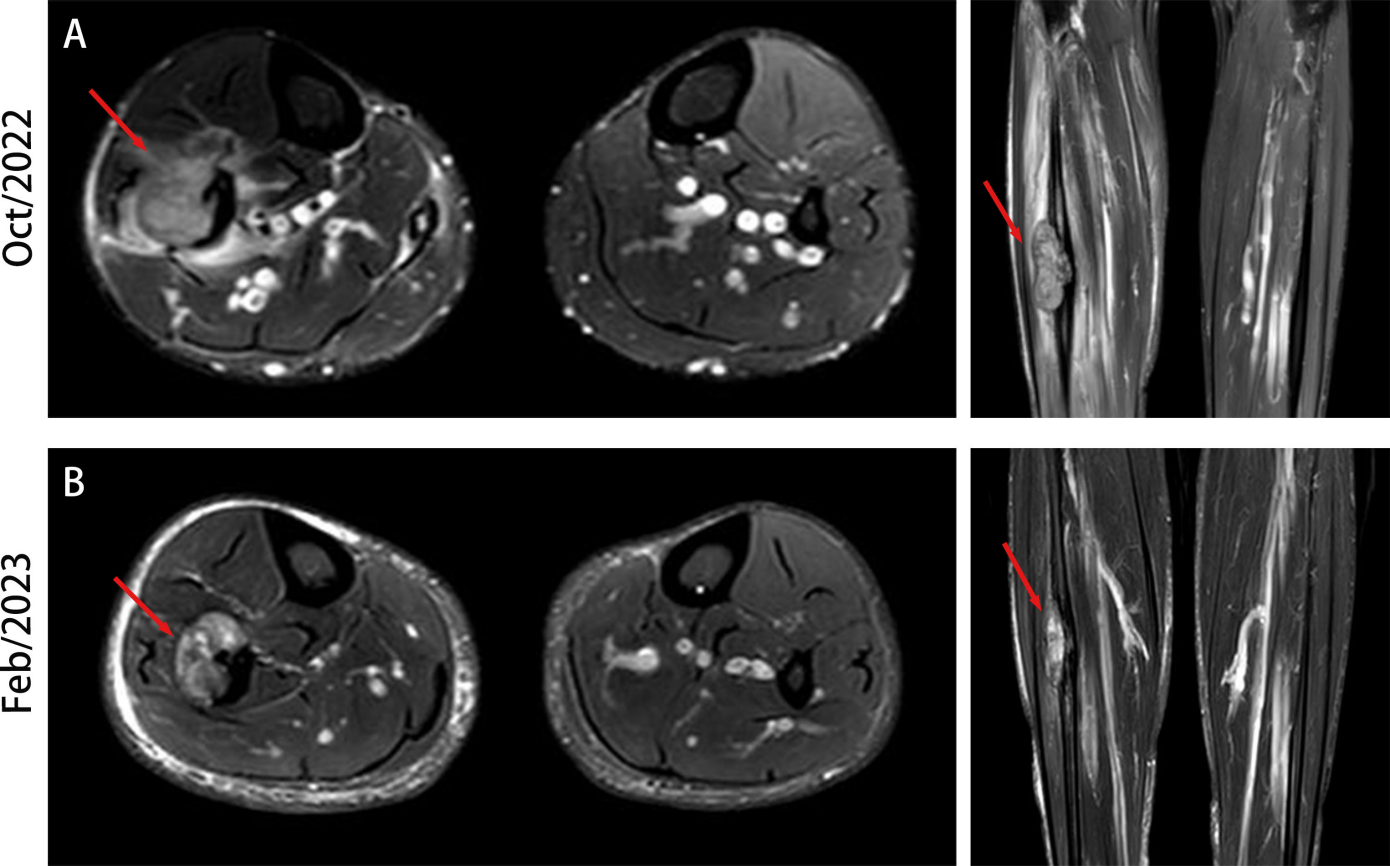
MRI of fibular lesion. **(A)** MRI highlights of fibular lesion at the time of initial diagnosis. **(B)** MRI presentation of fibular lesion after two cycles of treatment.

**Figure 2:**
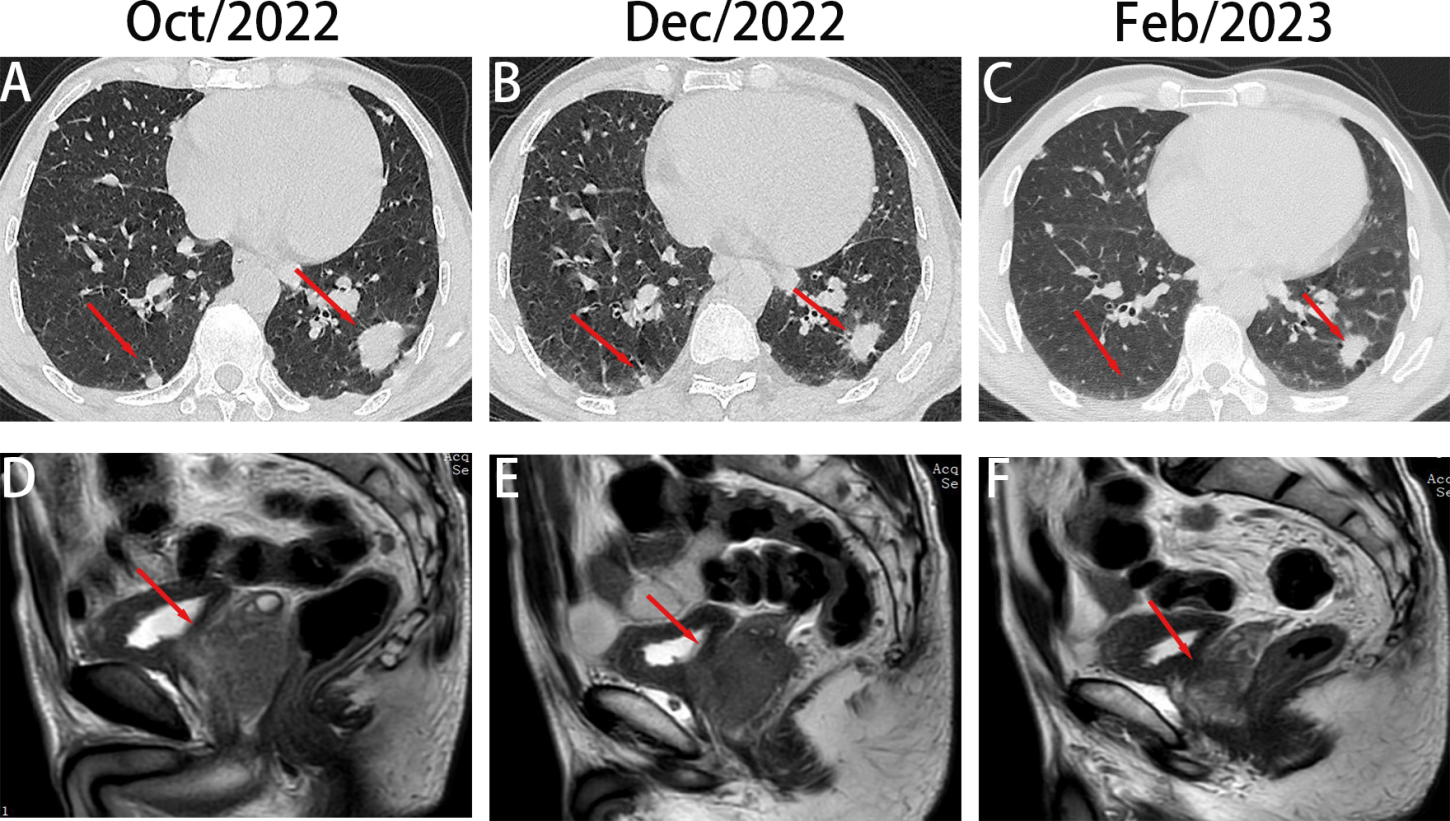
Chest computed tomography (CT) and prostate magnetic resonance imaging (MRI) scan of the patient throughout the course of diagnosis and treatment. **(A–C)**, Imaging changes of chest CT (mediastinal window and pulmonary window) and prostate MR at different time points **(D–F)**.

**Figure 3:**
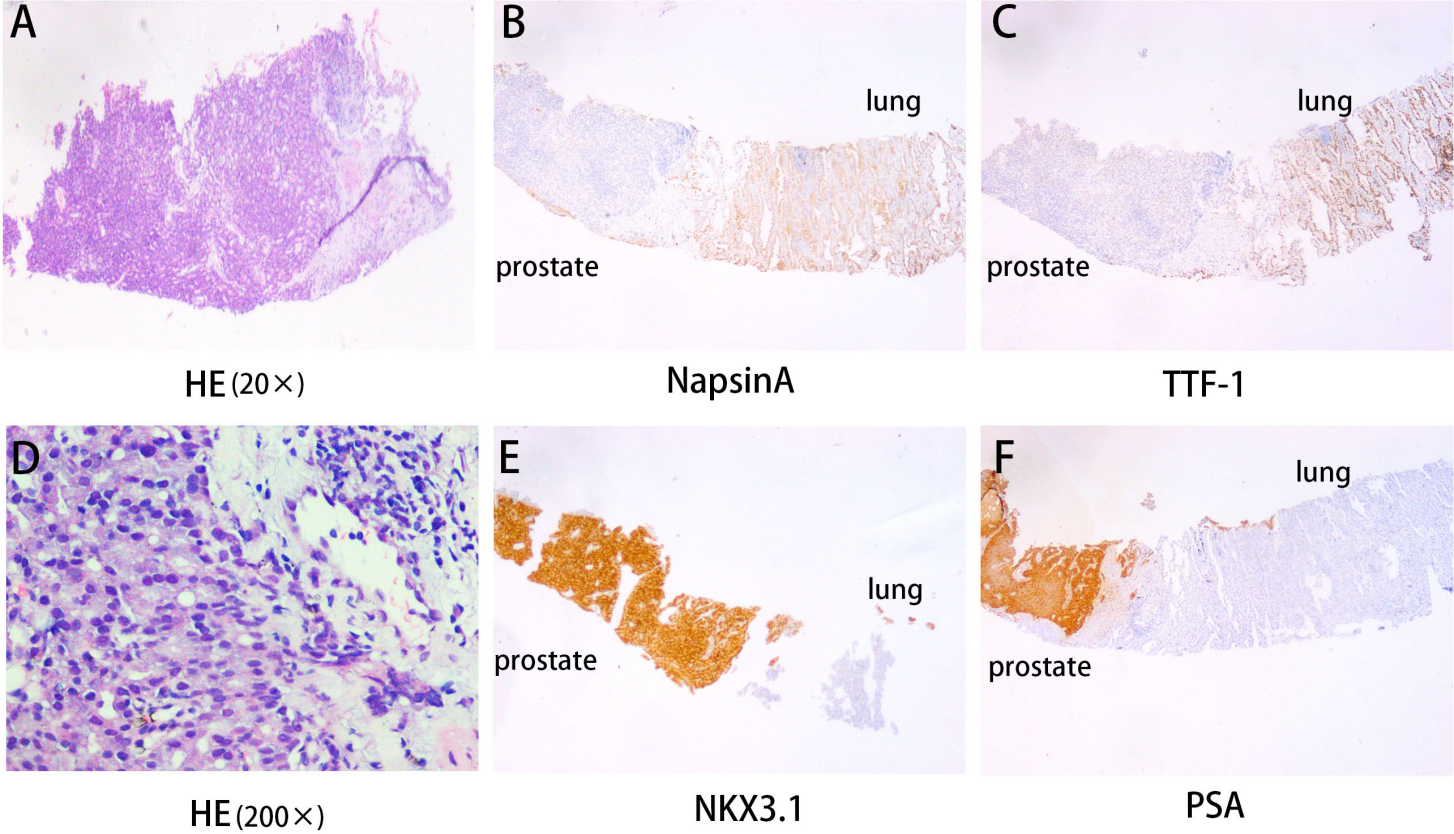
Lung puncture pathology results. **(A, D)** HE revealed lung adenocarcinoma and prostate cancer histology (20×) and (200×). **(B, C, E, F)** The immunohistochemical examination indicated malignant cells immunoreactive for NapsinA **(B)**; TTF-1 **(C)**; NKX3.1 **(E)** and PSA **(F)**. Magnification 100×.

A 58-year-old man presented to our hospital with a 1-month history of right lower limb pain. At physical examination, a lump was observed on the outer side of his left lower leg without signs of any inflammation. The patient was referred to our Department of Orthopedics. Magnetic resonance imaging (MRI) revealed an occupying lesion in the middle fibular segment, suggesting a tumor (**Figure 1A**). Subsequent chest computed tomography (CT) presented an occupying lesion in the lower lobe of the left lung, multiple small nodules in both lungs, and enlarged lymph nodes in the bilateral hilar mediastinum (**Figure 2A**), indicating metastatic tumors or multiple metastases. Subsequently, the patient underwent CT-guided percutaneous biopsy of the left lung mass. The histopathological examination revealed the presence of two distinct tumor cell components in the same paraffin block specimen: one composed of lung adenocarcinoma cells and the other composed of prostate adenocarcinoma cells. The results of the hematoxylin and eosin (HE) staining revealed the presence of two distinct tumor cell types in the same field of view (**Figures 3A, D**). The immunohistochemistry (IHC) staining provided an overview of both lung adenocarcinoma and prostate cancer metastasis. Within the same field of view, lung adenocarcinoma was predominantly observed on the left side of the image, while prostate cancer was mainly located on the right side. Additionally, notable differences in the expression of TTF-1, Napsin A, NKX3.1, and PSA were observed between the two tumor types (**Figures 3B, C, E, F**). Genetic testing is recommended for advanced lung adenocarcinoma to guide targeted therapy. The results of genetic testing revealed a deletion mutation in exon 19 of *Epidermal Growth Factor Receptor* (EGFR).

Further, MRI evaluation and PSA tests confirmed a prostate mass invading the seminal vesicle and posterior bladder wall, forming a soft tissue mass protruding into the bladder cavity (**Figure 2D**). The anterior rectal wall was also likely invaded, with metastatic lymph nodes in the bilateral inguinal region and the acetabulum. The total PSA level was >100 ng/mL.

**Figure 4:**
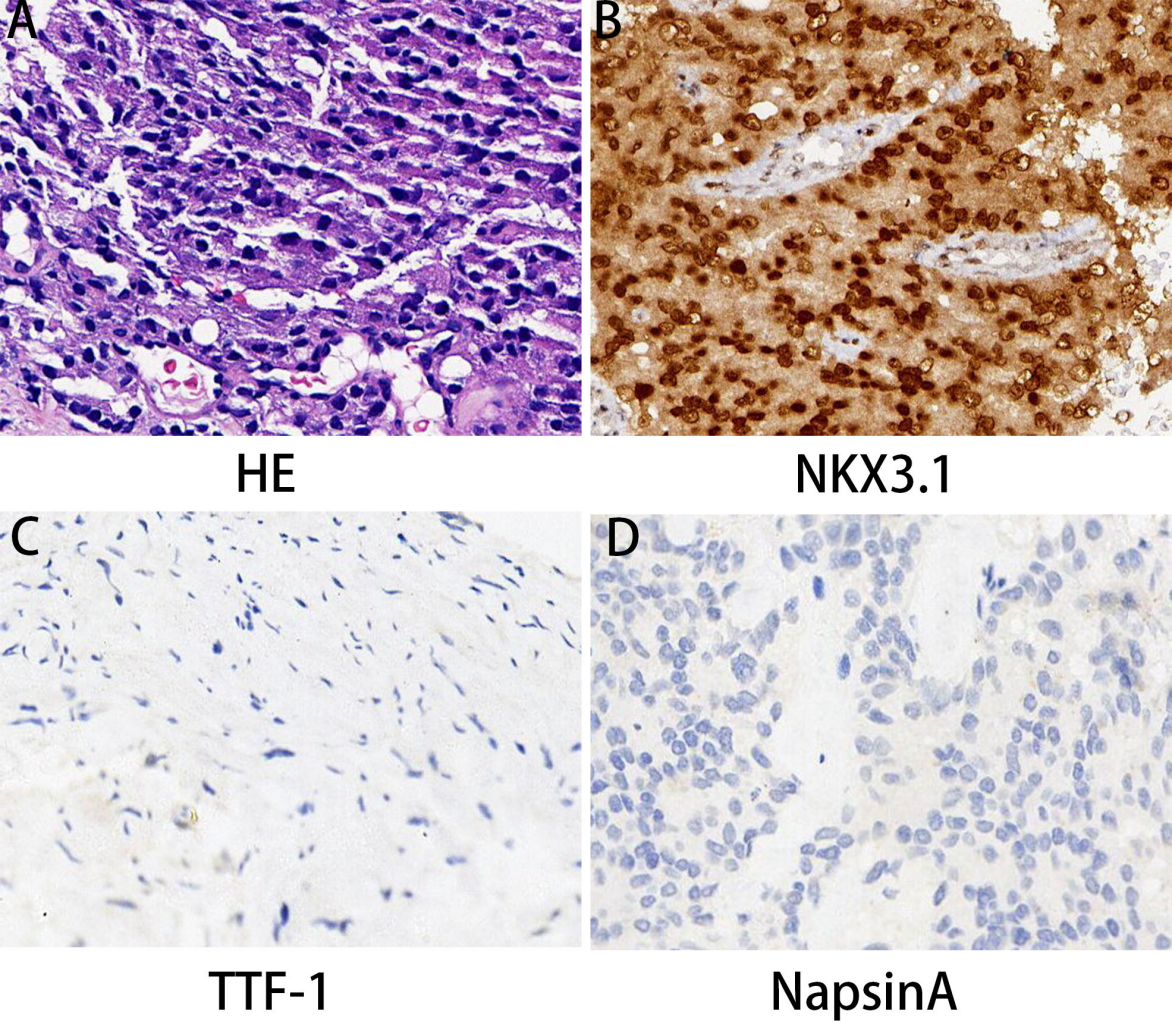
Prostate biopsy pathology results. **(A)** HE revealed prostate cancer histology. **(B–D)** The immunohistochemical examination indicated malignant cells immunoreactive for NKX3.1 **(B)** and negative expression for TTF-1 **(C)** and NapsinA **(D)**. Magnification 100×.

Percutaneous biopsies were performed on the prostate and fibular masses under CT guidance. Along with the aforementioned imaging results, pathological analysis revealed that the prostate mass was a prostatic acinar adenocarcinoma. The Gleason score on diagnosis was 8 (4 + 4 = 8/International Society of Urological Pathology Grade Group 4). HE staining also confirmed prostatic acinar adenocarcinoma (**Figure 4A**). According to the IHC results, the cancer cells were positive for NKX3.1 and Ki-67 (30%), whereas negative for TTF-1 and Napsin A (**Figures 4B–D**). A puncture was made on the fibula mass. HE staining revealed an adenoid growth pattern, as well as heterogeneous characteristics and nuclear atypia typical of lung adenocarcinoma (**Figure S1A**). IHC results showed that the cancer cells were positive for CK7, TTF-1, and NapsinA, but negative for PSA and NKX3.1 (**Figures S1B–F**). The Ki-67 positivity rate was 70%, which indicated metastatic lung adenocarcinoma. Furthermore, to provide a more comprehensive assessment of bone metastasis, we have refined the results of the Tc-99 m bone scintigram. It reveals abnormal metabolic activity in the lower cervical spine, left 9th rib articulation, 8th thoracic vertebra, and upper segment of the right tibia (**Figure S2**).

Based on the aforementioned results, the patient was diagnosed with left lower lobe adenocarcinoma (cT3N2M1c) and prostatic acinar adenocarcinoma (T4N1M1c), presenting a unique phenomenon of concurrent double primary cancers involving the prostate and lungs, along with the remarkable occurrence of prostate cancer metastasizing to the lungs.

On November 13, 2022, radiotherapy was initiated for the right fibular metastasis (planned dose: 36 Gy in 12 fractions). The patient also received targeted therapy with EGFR TKI amitinib and endocrine therapy with bicalutamide and leuprorelin. He underwent local radiotherapy for the fibular lesion. On December 14, 2022, chest CT revealed that the lesion in the lower lobes of the left and right lungs had shrunk compared with that observed on the previous scans (**Figure 2B**). Prostate MRI also presented a reduction in tumor size (**Figure 2E**). The PSA level decreased to 98.26 ng/mL, indicating that the treatment was effective. The patient continued to receive targeted and endocrine therapies. On February 13, 2023, upon re-examination with CT, the nodule in the lower lobe of the left lung had slightly decreased compared with that before, and the nodule in the right lung had significantly decreased, almost reaching complete remission (**Figure 2C**). Prostate MRI indicated that the size of the tumor had further reduced (**Figure 2F**). On MRI, the size of the fibular mass had decreased compared with that before (**Figure 1B**). The PSA level had declined further to 13.79 ng/mL, indicating that the treatment remained effective. The patient is currently receiving maintenance treatment with a combination of targeted and endocrine therapies. No disease progression or recurrence has been observed based on the current situation.

## Discussion

We report a case of concurrent double primary adenocarcinomas of the prostate and lung. This is the first report on prostate cancer metastasizing to lung adenocarcinoma, and thus, our findings may offer a reference for more TTM cases.

TTM refers to the growth and spread of a primary malignant tumor into an existing tumor through blood vessels or lymphatic pathways (7). TTM typically occurs in immunocompromised patients, such as organ transplant recipients or those with HIV (8). However, according to reports, the most common TTM-associated tumors include thyroid papillary carcinoma, renal cell carcinoma, melanoma, ovarian cancer, colorectal cancer, breast cancer, and prostate cancer (2). The diagnostic criteria were described by Campbell et al. (9) for TTM are as follows: (1) the presence of two or more distinct primary tumors; (2) the recipient tumor must be a true neoplasm; (3) the metastatic neoplasm is a true metastasis with established growth in the recipient tumor and not formed due to embolization or contiguous growth; and (4) exclusion of metastasis to lymphatic tissue already involved by lymphoreticular tumors. TTM is rare, and therefore, its diagnosis is challenging, and careful evaluation and differential diagnosis are required. No specific research or reports are currently available regarding the mechanism underlying the metastasis of prostate cancer to lung cancer. According to the existing literature, metastasis of prostate cancer to the lung may be related to the lung microenvironment, including lung vascular networks and immune cells (10–12). In addition, inflammation and lung injury may offer an environment suitable for the growth of prostate cancer cells (13, 14). Conjugate gene expression may also be a key player in metastasis of prostate cancer to the lungs (15). Two or more genes encoded within the same DNA fragment are termed conjugate genes. These genes often highly depend on each other in transcription and expression (16). In the lungs, prostate cancer cells may use activated conjugate gene pathways to enhance their survival and proliferation abilities.

Currently, reports of prostate cancer metastasizing to lung cancer are lacking, and thus, the treatment standard has not been established. The treatment strategy for dual primary cancers of the prostate and lung involving prostate cancer metastasis to the lung should be individualized based on specific conditions. The present patient with stage IV lung adenocarcinoma harboring an *EGFR* mutation received first-line targeted therapy according to the National Comprehensive Cancer Network guideline. Considering the patient also had prostate cancer, first-line hormonal therapy was administered for concurrent treatment. Of note, we need to focus on whether synergistic effects or antagonistic effects are present between the two tumor types during treatment, which may cause certain side effects to patients. Furthermore, local radiotherapy was added to achieve a better local control. So far, the therapeutic effect has been satisfactory. We believe that this step-by-step treatment model is feasible for TTM treatment.

There are certain limitations in our study. The follow-up time is relatively short. Although the current treatment outcomes appear promising, the treatment effects still require long-term therapeutic observation. Additionally, due to the lack of surgical indications, incomplete specimens, and the absence of specific imaging findings, we were unable to confirm the distribution of the tumor and metastasis. We will do our best to accumulate more similar cases to further clarify this situation.

In conclusion, we here report a rare and intriguing case of prostate cancer metastasizing to lung cancer, which enriches the literature on TTM. Our experience may also provide some insight on TTM treatment. The definite mechanism of prostate cancer metastasis to lung cancer is still not well understood. Further research is required to formulate effective treatment strategies.

## Data availability statement

The original contributions presented in the study are included in the article/**Supplementary Material**. Further inquiries can be directed to the corresponding author.

## Ethics statement

This study was approved by the Ethics and Scientific Committee of Hubei University of Medicine with approval number 2022PR-H002. Written informed consent was obtained from the individual for the publication of any potentially identifiable images or data included in this article. The patients/participants provided their written informed consent to participate in this study.

## Author contributions

Conceptualization, DZ; data curation and writing, writing—review and editing, SL, YD, and HL; funding acquisition, DZ and SL. All authors have read and agreed to the published version of the manuscript.

## Supplementary material

Supplementary Figure 1 Fibular mass biopsy pathology results. **(A)** HE revealed prostate cancer histology. **(B–F)** The immunohistochemical examination indicated malignant cells immunoreactive for TTF-1 **(B)**; strongly positive NapsinA **(C)**; moderately positive for CK7 **(D)**; negative expression for NKX3.1**(E)** and PSA **(F)**. Magnification 100×.

Supplementary Figure 2 Bone scintigraphy.
