# Supplementary figures and images for: Case Report: Tumor-to-tumor metastasis with prostate cancer metastatic to lung cancer: the first reported case
Source: Front Oncol. 2023 Aug 18;13:1238331. doi: 10.3389/fonc.2023.1238331 (PMC10471885; doi:10.3389/fonc.2023.1238331)

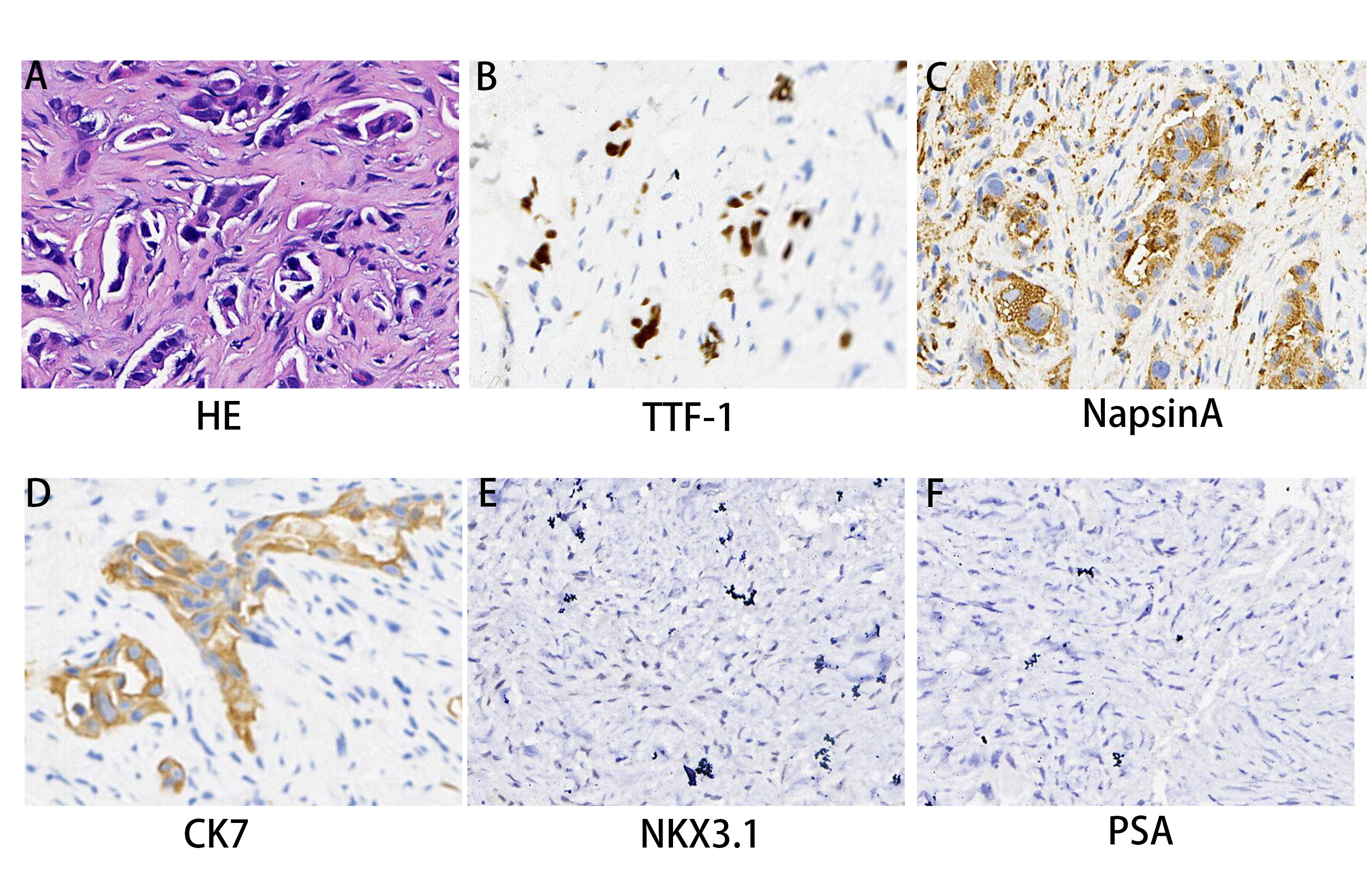

Supplement: Supplementary Figure 1 — Fibular mass biopsy pathology results. (A) HE revealed prostate cancer histology. (B–F) The immunohistochemical examination indicated malignant cells immunoreactive for TTF-1 (B); strongly positive NapsinA (C); moderately positive for CK7 (D); negative expression for NKX3.1(E) and PSA (F). Magnification 100×. [file Image_1.jpeg]

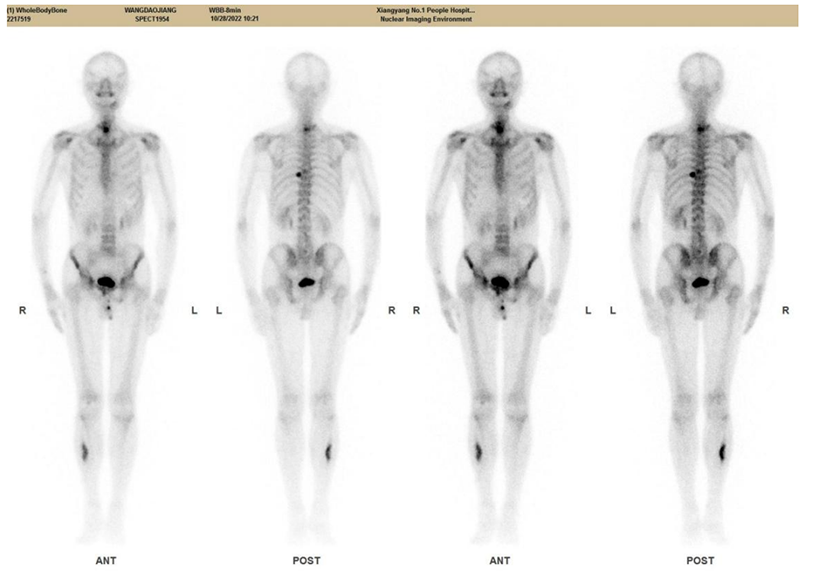

Supplement: Supplementary Figure 2 — Bone scintigraphy. [file Image_2.tif]
